# Biopsy proven lupus nephritis in a black male patient in West Africa with systemic lupus erythematosus: case report

**DOI:** 10.11604/pamj.2018.31.198.14326

**Published:** 2018-11-22

**Authors:** Elliot Koranteng Tannor, Kwame Yeboah-Mensah

**Affiliations:** 1 Department of Medicine, Komfo Anokye Teaching Hospital, Kumasi, Ghana

**Keywords:** Lupus nephritis, black male, West Africa, renal biopsy, systemic lupus erythematosus

## Abstract

*Lupus nephritis* (LN) is a complication of systematic *lupus erythematosus* (SLE) with significant impact on morbidity and mortality. It is known commonly to affect females but has worse prognosis when males or blacks are affected. In a setting with deficiency in health care delivery, the diagnosis and adequate management of SLE patients becomes difficult and hence less cases of *lupus nephritis* in black males have been reported. We present a case of a male black patient with SLE who presented with *neurolupus, serositis*, skin manifestations and Class IV/V *lupus nephritis* on histology. Our case highlights the challenges in the management of *lupus nephritis* and the complications with immunosuppression in an attempt to induce remission. There is the need for high index of suspicion for the diagnosis of *lupus nephritis* especially in black males for prompt management to get the best outcome.

## Introduction

Systemic *lupus erythematosus* (SLE) is a multi-systemic autoimmune condition with significant morbidity and mortality especially when it affects the kidneys. *Lupus nephritis* (LN) is recognised as a serious complication in SLE patients. It is second only to infection as the most common cause of mortality in SLE patients. In Africa, the prevalence of LN among SLE patients is 49 - 60% [1]. SLE tends to affect females predominantly but males tends to have more severe disease with poorer prognosis. Renal survival in SLE patients among blacks is poor especially with diffuse proliferative *nephritis* on biopsy and with impaired renal function [2]. We set out to report a case of *lupus nephritis* in a black male patient with class IV/V *lupus nephritis* on renal histology who presented with hypertension, renal impairment, nephrotic range proteinuria, high ANA and anti-SM titre. Patient is currently on immunosuppression in partial remission but has experienced complications related to the use of immunosuppression.

## Patient and observation

A 22-year-old black male patient with a two year history of recurrent maculopapular rashes on the face, limbs and abdomen being seen by a dermatologist with a suspicion of SLE and occasional joint pains on methotrexate, steroids and hydroxychloroquine, presented to the emergency unit with a two week history of bilateral pedal edema, oliguria, haematuria, abdominal distension and scrotal swelling and a single episode of generalized tonic-clonic seizure. He was noted to be pale, afebrile with a temperature of 36.3° Celsius, a malar rash with multiple hyperpigmented macular rashes all over the body. His hair was thin and brownish with periorbital edema and dark nails. He had hypertension with a blood pressure of 156/100mmHg and a pulse of 117 beats per minute which was regular with good volume. Chest had bilateral basal crackles and a stony dull percussion note on the left hemi-thorax with decreased vocal resonance. He was conscious and oriented with no obvious neurological deficits with power of 5/5 in all limbs when examined. His hypertension was managed with furosemide 80mg three times a day, nifedipine 30mg daily and carvedilol 12.5mg twice a day. He was haemotransfused one unit of blood. His laboratory investigations revealed hemoglobin level of 7.4g/dl, serum urea of 23.7mmol/L and serum creatinine of 256umol/L which rose to a peak of 433μmol/L on admission associated with decreasing urine output of between 300 - 600ml/day. He also had hypercholesterolemia (14.5mmol/L) and hypoalbuminemia (13.1g/L). He had haematuria (+) and urine protein on dipstick was (++++) with urine protein creatinine ratio (UPCR) of 6.29 g/day. The serum anti-nuclear antibody (ANA) was positive with a titre of 1/160 and the anti-smith (SM) antibody was greater than 480U/ml but the anti-double stranded deoxyribonucleic acid antibody (anti-dsDNA) was negative. Renal ultrasound revealed echogenic but enlarged kidneys measuring 13.2 x 5.3cm on the right and 13.1 x 8.0cm on the left with poor cortico-medullary differentiation. No calculi, hydronephrosis or masses were noted. There was moderate ascites and left pleural effusion on ultrasound.

He was then worked up for a percutaneous renal biopsy which was done under ultrasound guidance by the attending nephrologist as there were no contraindications. He was discharged the next day to report with the histology report on outpatient basis. Histology report revealed diffuse proliferative with mesangial expansion and increased cellularity. The capillaries appeared to have thickened walls and the lobules appeared accentuated. There were no crescents, necrosis or thrombi. The *intestitium* showed edema, areas of necrosis and associated with chronic active inflammation. The tubules were focally dilated and with hyaline eosinophilic luminal contents. These findings were suggestive of *lupus nephritis* class IV with elements of class V LN. He was made to continue the high dose prednisolone of 60mg/day and mycophenolate mofetil (MMF) of 1.5g twice a day and followed up monthly on outpatient basis with proteinuria, serum albumin, full blood count and renal functions results. There was marked improvement of the renal function with good urine output and reduction in serum creatinine to 95umol/L after six months of treatment. There was also improvement of the serum albumin from 13g/L to 36g/L and total cholesterol also decreased to 7.1mmol/L from 14mmol/L after six months of treatment. His hemoglobin level also improved to 13.5 g/dl from 7g/dL on admission after only a unit of whole blood was transfused.

However, he developed acne after one month on steroids and also an extended spectrum beta lactamase (ESBL) Enterobacter aerogenes urinary tract infection (UTI) after three months of immunosuppression. The organism was sensitive to ertapenem, imipenem, meropenem, colistin and amikacin. It was resistant to amoxycilliin, clavulanic acid, ciprofloxacin, cotrimoxazole and nitrofurantoin. Amikacin was chosen due to its availability and the renal functions watched closely as amikacin is well known to be nephrotoxic. His UTI resolved with parenteral amikacin. He also developed oropharyngeal candidiasis with complaint of odynophagia and was treated with oral fluconazole for three days. He also developed herpes zoster opthalmicus affecting the trigeminal nerve distribution on the left side of the face which was also treated effectively with acyclovir. These infections led to the reduction of immunosuppression to enable the adequate treatment of the fungal, bacterial and viral infections due to the potent immunosuppression. The reduction in immunosuppression led to a marked increase in urine proteinuria creatinine ratio (UPCR) to 9.7g/day from 4.9g/day the previous month. He developed post herpetic neuralgia after the healing of the herpes zoster infection and was put on amitriptyline with very good response. He is currently being followed up at the renal clinic of the Komfo Anokye Teaching Hospital where he is now on increased dose of MMF 1gm twice daily, prednisolone 10mg daily, irbesartan 300mg daily, hydroxychloroquine 200mg a day from Monday to Friday, atenolol 50 daily, methyldopa 500mg three times a day, Tab Calcium/ Vitamin D 1g/800IU daily and atorvastatin 20mg every evening. He is in partial remission with a proteinuria that has markedly improved to 0.55g/day with no new complication from the medications now on maintenance therapy of MMF 500mg twice daily and prednisolone 5mg daily.

## Discussion

To our knowledge this is the first ever case report of a case of SLE in a black male patient with biopsy-proven *lupus nephritis* in Ghana. This report highlights the presentation and management in this rare demography as lupus has been shown to be more common in young females in their reproductive age. The patient responded well to immunosuppression but had several complications related to the immunosuppression used with the development of acne, herpes zoster ophthalmicus, ESBL Enterobacter aerogenes urinary tract infection and oropharyngeal candidiasis.

Systemic lupus erythromatosis (SLE) is a multi-system autoimmune condition. The prevalence of SLE has increased worldwide affecting young women in their reproductive prime with less prevalence among males. Some SLE cases have been reported in Africa and also in Ghana [3]. SLE is an autoimmune condition with diagnosis based on the American college of rheumatology (ACR) criteria which requires four out of eleven of the criteria for the diagnosis of SLE [4]. Our patient had arthritis, serositis, renal disorder, thrombocytopenia and neurolupus as he presented with a seizure. He also had a positive ANA and a very high anti-Smith antibody (Anti-SM) titre making 6 out of the 11 required criteria. The sensitivity of anti-SM antibody is low (24% -30%) but has high specificity of 96 - 98%. Anti-Smith antibodies are known to be more prevalent in black patients [5] as was found in our patient. Anti-Smith antibodies can confirm the diagnosis of SLE. Lupus nephritis (LN) has been scarcely described in Ghana and West Africa probably as a result of poor reportage and even much less cases of biopsy proven lupus nephritis. LN is a recognised complication of SLE patients; it is associated with poor outcomes and impacts significantly on morbidity and mortality. In Africa, the prevalence of LN among SLE patients is 49 - 60%. LN is the most common secondary cause of glomerulonephritis in a single centre study of the pattern of renal diseases accounting for 39% of cases in South Africa. LN is the second most common cause of mortality after infection in SLE patients [6].

Renal biopsy is essential for the diagnosis of LN, to classify the disease, assess severity, to prognosticate and exclude other causes of renal diseases in SLE patients [1]. The patient’s presentation with nephrotic range proteinuria, hypertension, renal impairment, hypoalbuminuria and hypercholesterolemia were all clinically suggestive of lupus nephritis class IV or class V [7]. Histology however proved Class IV LN with thickened capillary walls suggestive of elements of Class V lupus nephritis but could not be confirmed on electron microscopy as the only stain available in our setting was the Hematoxylin and Eosin (H and E) stains as a low resourced center. Immunofluorescence and electron microscopy were not available in our setting. This is a challenge as light microscopy alone is inadequate for the proper diagnosis of lupus nephritis. There may also have been an element of interstitial nephritis as there was edema and chronic active inflammation with eosinophils on histology report as has been described in up to 50% of cases of class IV LN in a study. Class IV LN is the commonest presentation of lupus nephritis and very aggressive when not well managed. It tends to present with sub-nephrotic proteinuria but class V membranous glomerulonephritis tends to present with nephrotic range proteinuria as noted in our patient with a peak as high as 9.7g/day [7].

Cyclophosphamide (intravenous or oral) or mycophenolate mofetil (MMF) with high dose steroids have been used for the treatment of proliferative nephritis. MMF has been found to be comparable to intravenous cyclophosphamide but with less side effects [8]. MMF was used in our patient for induction with high dose oral steroids. Response to immunosuppression was good with improvement of renal function and a reduction of proteinuria from a peak of 9.7g/day to 0.55g/day after six months of induction. This improvement in renal function and proteinuria was not without side effects. Challenges ranged from high cost of medications to patient and complications with the use of mycophenolate mofetil and high dose prednisolone.

He reported with facial acne about a month after the initiation of steroids which resolved on tapering of the steroids. Acne has been shown to be a complication of steroid use. He also then developed steroid induced dyspepsia which also necessitated the rapid tapering of the prednisolone from 60mg a day to 40mg a day and oral proton pump inhibitor initiated with good clinical response. Steroid use causes of dyspepsia in high doses and have been well described. Bacterial infections have been reported in SLE patients on immunosuppression. This has been attributed to complement deficiency, disease activity, renal impairment, use of glucocorticoids and cytotoxic agents such as MMF as used in our patient [9]. The patient developed extended spectrum beta lactamase (ESBL) Enterobacter aerogenes urinary tract infection which was effectively treated with antibiotics. Urinary tract infections are not common in males but the use of MMF and prednisolone increased the risk of infection in patients with autoimmune conditions such as SLE [10]. Oropharyngeal candidiasis have also been described in systemic lupus erythematosus patients and the use of immunosuppression [9, 10]. The development of oropharyngeal candidiasis complicates treatment and may impair intake of food and oral medications. There was good response to oral fluconazole which is indicated in the treatment of candidiasis and found to be superior to nystatin in patients with immunosuppressive disease like SLE.

There is increased incidence of herpes zoster reactivation associated autoimmune conditions including SLE and the use of high dose MMF has been associated with herpes zoster infection. Herpes zoster has been shown to be the most common infection among SLE patients on immunosuppression [10]. Despite the challenges expected in a black male patient with lupus nephritis class IV/V LN and the numerous complications as a result of the disease process and or the immunosuppression treatment, our patient is in partial remission and doing very well clinically.

## Conclusion

Lupus nephritis has been less described among black males though known to be aggressive. Its management is associated with fungal, viral and bacterial infections. Prognosis is associated with Class of lupus nephritis, renal functions and complications of SLE. In our setting with inadequate facilities and poor patients who cannot afford investigations and treatment, procedures and treatment of lupus and lupus nephritis is a nightmare for the physician, rheumatologist and nephrologist. A high index of suspicion is required for the diagnosis of lupus nephritis in black males and a percutaneous renal biopsy imperative in the management.

## Competing interests

The authors declare no competing interests.
